# Cystic Fibrosis, New Frontier: Exploring the Functional Connectivity of the Brain Default Mode Network. Comment on Elce et al. Impact of Physical Activity on Cognitive Functions: A New Field for Research and Management of Cystic Fibrosis. *Diagnostics* 2020, *10*, 489

**DOI:** 10.3390/diagnostics11061001

**Published:** 2021-05-31

**Authors:** Simone Gambazza, Rita Maria Nobili, Riccardo Biffi, Paul Eugene Summers, Carla Colombo, Antonella Costa

**Affiliations:** 1 Cystic Fibrosis Centre, Fondazione IRCCS Ca’ Granda Ospedale Maggiore Policlinico, 20122 Milano, Italy; ritamaria.nobili@policlinico.mi.it (R.M.N.); carla.colombo@unimi.it (C.C.); 2 Direzione delle Professioni Sanitarie, Fondazione IRCCS Ca’ Granda Ospedale Maggiore Policlinico, 20122 Milano, Italy; 3 Department of Neuroradiology, Fondazione IRCCS Ca’ Granda Ospedale Maggiore Policlinico, 20122 Miano, Italy; rickysax@gmail.com (R.B.); pefs44@yahoo.co.uk (P.E.S.); antonella.costa@policlinico.mi.it (A.C.); 4 Department of Pathophysiology and Transplantation, Università degli Studi di Milano, 20122 Milano, Italy

We read with great interest the paper entitled “Impact of physical activity of cognitive functions: a new field for research and management of Cystic Fibrosis” by Elce et al. [1]. Physical activity (PA) was described as a non-pharmacological intervention found to be associated not only with important clinical outcomes, such as survival, but also with cognitive abilities, thus suggesting this field of investigation as a potential leverage to optimize the management of cystic fibrosis (CF). They provided a comprehensive summary of the evidence regarding the effects of PA on neurocognitive functions expected in patients with CF. 

Considering the broad expression of the Cystic Fibrosis Transmembrane Conductance Regulator (CFTR) protein in the adult nervous system [2,3,4,5], and the available findings from neuroimaging studies on chronic obstructive pulmonary disease (COPD), which is characterized by specific patterns of moderate-to-severe deficits in higher neuronal and complex visual-motor processes [6,7], in 2016, our multidisciplinary team started investigating the functional connectivity of patients with CF, combining neuropsychological assessment and resting-state functional magnetic resonance imaging (rs-fMRI) with the results of exercise testing.

The notion of rest as a passive state has been challenged by functional neuroimaging studies showing that sets of brain regions display coherent activity at rest [8]. One such network, comprising the medial pre-frontal cortex (MPFC), posterior cingulate cortex (PCC), precuneus, inferior parietal and lateral temporal cortices, has a topographical consistency across subjects that has led some authors to posit the existence of an active, organized, baseline mode of brain function that would constitute a fundamental default mode network (DMN) of brain function [9,10]. The widely distributed brain areas that compose the DMN include regions associated with both cognitive and affective functions, though it is increasingly recognized that traditionally defined areas often overlap the boundaries of brain architectonic areas [11].

The functional connectivity of DMN sub-regions via a seed-based correlation analysis of rs-fMRI data was investigated in 11 adult subjects with a confirmed diagnosis of CF homozygous for the Phe508del mutation. Balancing administration time and the need for a sensitive evaluation in a population known as cognitively unaffected, neuropsychological evaluation (NPE) covered six higher-function cognitive-domains: attention, memory, executive functions, language, perceptive and praxis. Furthermore, to evaluate exercise capacity, an incremental cycling protocol to volitional fatigue was performed without metabolic measurement [12], as described elsewhere [13]. 

The functional connectivity between each seed region and other brain areas showed significant dependencies on exercise capacity and NPE components in the second-level rs-fMRI analysis after FDR-correction, suggesting that modulation of these connections is associated with exercise, the neuropsychological components, or both. Using the MPFC as the seed (Figure 1), functional connectivity with the frontal medial cortex, the left paracingulate gyrus and the subcallosal cortex showed the largest difference between normal and abnormal exercise responders, while accounting for the effects of two neuropsychological components, summarized as working memory and depression and/or anxiety, and the subjective perception of health and the visuo-spatial functions.

Similarly, the precuneus, the posterior cingulated gyrus, and DMN regions parietal lobe (PL)-right and PL-left showed the largest differences in functional connectivity with the PCC seed. The F-values for connectivity differences to the PCC tended to be greater than those seen for the other seeds, with the differences in connectivity between the PCC and the precuneus having the largest F-values encountered. For PL-left seeding, the lateral occipital left and other components of the DMN (specifically PL-right and PCC) showed the largest effect, while for PL-right seeding the largest differences were seen in the DMN regions PL-left and PCC, the right lateral occipital cortex, the precuneus and the left lateral occipital.

Our preliminary data document a significant association between the posterior cingulate and MPFC. There is a consensus in the literature concerning the existence of an anterior and a posterior part of the DMN, each having an area that may be termed its main hub. The MPFC is the anterior hub, and the posterior cingulate cortex—precuneus (PCC/Precuneus) in the posterior DMN [14]. Anterior regions of the network such as the medial prefrontal cortex have been associated with self-referential processing [15], and participate in close functional coupling between MPFC and PCC [16].

The connectivity of the MPFC correlated with the exercise capacity and NPE components and included frontal medial cortex, left paracingulate gyrus and subcallosal cortex, all of which belong to the executive control network [17]. The human inferior PL-left plays a pivotal role in many cognitive functions [18]. 

The present findings also suggest a particularly strong effect of exercise and NPE domains on the connectivity of the PCC and precuneus, with the PCC showing a greater association with exercise response and NPE components than we found for the other seed regions. The PCC and precuneus are major nodes within the DMN and have high metabolic activity and dense structural connectivity to widespread brain regions, which suggests that it has a role as a cortical hub [19]. Previous studies demonstrated the involvement of the precuneus in the manipulation of mental images and in internally guided attention derived from visuo-spatial imagery studies; these findings implicate its unique capacity in mental representation of the internal self [20]. These results are consistent with this notion, considering the involvement of the precuneus in the altered functional connectivity of both the PCC and PL-right. Although we cannot presently describe the mechanisms determining the observed modulation of connectivity, our findings suggest a relation between exercise and neuropsychological functions in patients with CF at the level of brain connectivity that is worth further exploration. If confirmed by larger and more heterogenous studies, these findings could provide interesting food for thought concerning daily clinical practice. For example, the existence of a linkage between emotions and the higher functions, both necessary to carry out a complex daily care plan such as that for the treatment of CF, might suggest the importance of ensuring that patients have enough time to memorize long lists of information and therapies, because it will likely influence adherence. Also, a higher level of exercise capacity may translate not only into a life-prolonging effect, but also into a neurocognitive enhancement advantage. In CF, very few studies have focused on neurocognitive or neuropsychological aspects combined with PA. The recent paper by Gold et al. [21] reported interesting results involving a mixed cohort of children with and without CF undergoing lung transplantation, whereas our sample included adults in a very narrow range of age, linking together PA, neuropsychological data and brain connectivity.

To the best of our knowledge, this report on a small cohort is the first to illustrate characteristics of rs-fMRI connectivity in individuals with CF. The observed modulation of brain networks under resting-state conditions may pave the way for future investigations aimed to support the relevance of physical activity for the well-being of persons with CF and the need for a through-targeted neuropsychological evaluation. As Elce et al. concluded in their review, the effects of exercise on mental health represent a promising and unexplored research field in CF. As a low-cost intervention, physical activity would be important not only in relation to lung health, but also to the cognitive functions that drive the choices we make in everyday life.

## Figures and Tables

**Figure 1:**
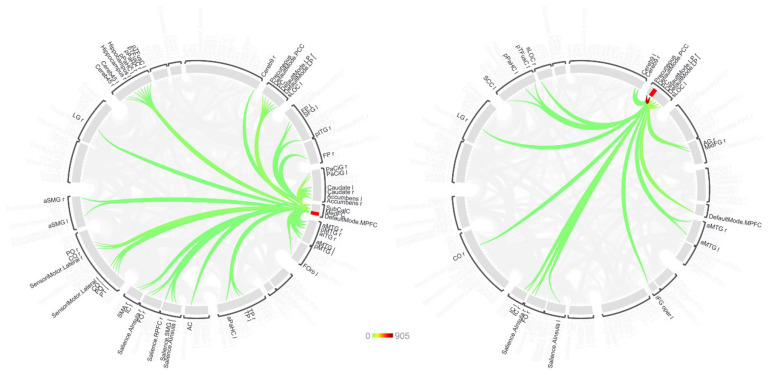
Results of second-level rs-fMRI analysis for seeds in the MPFC and PCC (left and right, respectively) displayed as a connectome ring. Around the ring, 165 brain regions are indicated that were evaluated for connectivity with the seed in the first-level analysis. The coloured traces represent connections that were seen in second-level analysis to be significantly associated with a combination of exercise performance and first principal component of the neuropsychological evaluation results after corrected for multiple comparison (FDR, *p* < 0.05). The rings were plotted with a common scale (coloured bar) based on the largest F-statistic encountered in this cohort (PCC-precuneus).

## Data Availability

The data presented in this study are available on request from the corresponding author.
